# Characterization and Biomarker Analyses of Post-COVID-19 Complications and Neurological Manifestations

**DOI:** 10.3390/cells10020386

**Published:** 2021-02-13

**Authors:** Bing Sun, Norina Tang, Michael J. Peluso, Nikita S. Iyer, Leonel Torres, Joanna L. Donatelli, Sadie E. Munter, Christopher C. Nixon, Rachel L. Rutishauser, Isabel Rodriguez-Barraquer, Bryan Greenhouse, John D. Kelly, Jeffrey N. Martin, Steven G. Deeks, Timothy J. Henrich, Lynn Pulliam

**Affiliations:** 1 Department of Laboratory Medicine, San Francisco VA Health Care System, San Francisco, CA 94121, USA; Bing.sun@va.gov (B.S.); Norina.Tang@va.gov (N.T.); 2 Division of HIV, Infectious Diseases, and Global Medicine, Department of Medicine, University of California at San Francisco, San Francisco, CA 94110, USA; Michael.peluso@ucsf.edu (M.J.P.); Rachel.rutishauser@ucsf.edu (R.L.R.); Isabel.rodriguez@ucsf.edu (I.R.-B.); Bryan.greenhouse@ucsf.edu (B.G.); Steven.deeks@ucsf.edu (S.G.D.); 3 Division of Experimental Medicine, Department of Medicine, University of California at San Francisco, San Francisco, CA 94110, USA; nikita.iyer@ucsf.edu (N.S.I.); Leonel.Torres@ucsf.edu (L.T.); Joanna.donatelli@ucsf.edu (J.L.D.); Sadie.munter@ucsf.edu (S.E.M.); Christopher.nixon@ucsf.edu (C.C.N.); Timothy.henrich@ucsf.edu (T.J.H.); 4 Department of Epidemiology and Biostatistics, University of California at San Francisco, San Francisco, CA 94158, USA; Dan.kelly@ucsf.edu (J.D.K.); Jeffrey.martin@ucsf.edu (J.N.M.); 5 Department of Laboratory Medicine and Medicine, University of California at San Francisco, San Francisco, CA 94143, USA

**Keywords:** SARS-CoV-2, neurodegeneration, exosome, cytokines, comorbidities

## Abstract

As the SARS-CoV-2 pandemic continues, reports have demonstrated neurologic sequelae following COVID-19 recovery. Mechanisms to explain long-term neurological sequelae are unknown and need to be identified. Plasma from 24 individuals recovering from COVID-19 at 1 to 3 months after initial infection were collected for cytokine and antibody levels and neuronal-enriched extracellular vesicle (nEV) protein cargo analyses. Plasma cytokine IL-4 was increased in all COVID-19 participants. Volunteers with self-reported neurological problems (nCoV, *n* = 8) had a positive correlation of IL6 with age or severity of the sequalae, at least one co-morbidity and increased SARS-CoV-2 antibody compared to those COVID-19 individuals without neurological issues (CoV, *n* = 16). Protein markers of neuronal dysfunction including amyloid beta, neurofilament light, neurogranin, total tau, and p-T181-tau were all significantly increased in the nEVs of all participants recovering from COVID-19 compared to historic controls. This study suggests ongoing peripheral and neuroinflammation after COVID-19 infection that may influence neurological sequelae by altering nEV proteins. Individuals recovering from COVID-19 may have occult neural damage while those with demonstrative neurological symptoms additionally had more severe infection. Longitudinal studies to monitor plasma biomarkers and nEV cargo are warranted to assess persistent neurodegeneration and systemic effects.

## 1. Introduction

The novel coronavirus SARS-CoV-2 continues to infect millions of individuals worldwide. While symptoms of COVID-19 are primarily systemic or respiratory, neurological complaints include anosmia, ageusia, altered consciousness, headache, seizures, and paresthesias [1,2,3]. The first study to look at neurological signs of COVID-19 infection came from Wuhan, China, where they found 36% of hospitalized infected individuals had neurologic symptoms [4]. Studies on coronaviruses SARS-CoV-1 and MERS show that a subset of individuals do not return to normal health after infection and can experience a number of neuropsychiatric sequelae for years after acute infection, including memory loss, attention deficit, and slow processing speed [2]. The primary targets of the virus are the angiotensin-converting enzyme 2 (ACE2) receptor-enriched epithelial cells of the respiratory and gastrointestinal tracts [5]. Human neurons are known to have a low level of ACE2 receptors, which may support the neuroinvasive potential of SARS-CoV-2 [6]. However, neuroinvasion has not been consistently shown, as numerous studies report undetectable SARS-CoV-2 in cerebrospinal fluid (CSF) [7]. Several publications using human brain organoid cultures showed SARS-CoV-2 infects neural progenitor cells and neurons with release of infectious virus [8,9]. Both respiratory and neural routes can cause infection of the myeloid lineage capable of a robust inflammatory and potentially long-lasting cytokine response to virus infection. 

Long-term health problems, including neurological symptoms such as headache, fatigue, dizziness, memory loss, confusion, and difficulty focusing, are associated with post-COVID-19 infection [10]. Over 30% of post-COVID-19 individuals complained of memory loss [11]. As many of these individuals experience a myriad of physical, cognitive and mental issues post COVID, some investigators have suggested using a general functional scale for assessing symptoms or quality of life [12,13]. One study lists the top 3 debilitating symptoms of “Long COVID” as fatigue, malaise, and cognitive dysfunction [13]. 

Extracellular vesicles (EVs) are small microvesicles shed from all cells under normal and pathologic conditions [14]. By packaging host cell proteins, microRNAs, and nucleic acids, EVs selectively reflect the parent cell’s state at the time of secretion, either by releasing toxic waste products, signaling cell damage or acting as a protective mechanism. EVs are taken up by recipient cells, thereby maintaining homeostasis. Alternatively, the parent cell under stress may release EVs that are taken up by recipient cells, deleteriously altering them [15,16,17]. EVs can also diffuse across the blood–brain barrier (BBB) and into the periphery where they can be selectively captured using cell surface specific antibodies [18,19]. Neuronal-enriched EVs (nEVs) can be isolated using antibodies against the L1 cell adhesion molecule (L1CAM), which is expressed on neurons. nEVs likely reflect the state of the neuron in real time [18,19]. nEVs containing the neurotoxic proteins amyloid beta (Aβ), neurofilament light (NFL), p-T181-tau, and/or the inflammatory protein HMGB1 have been isolated from individuals with HIV cognitive impairment [20,21], Alzheimer’s disease (AD) [22], and traumatic brain injury (TBI) [23,24,25]. These nEV proteins may predict neurological pathologies years in advance, as reported in AD [22,26,27]. We have previously used nEVs as biomarkers for cognitive impairment in HIV and to differentiate HIV-associated cognitive impairment from AD [21,28]. Examining interactions at the cellular level may help elucidate the mechanisms behind these long-term symptoms. 

It was the purpose of this exploratory study to evaluate peripheral markers of inflammation associated with neurological dysfunction plus nEV cargo to explain the post-COVID-19 neurocognitive symptoms in the early stage of post-COVID-19 recovery. We analyzed plasma from 24 post-COVID-19 volunteers from the Long-term Impact of Infection with Novel Coronavirus (LIINC) cohort that was established at the University of California, San Francisco [29]. These included individuals with neurological complaints (nCoV) and those with other lingering post-COVID complaints (CoV). We looked at several clinical variables that might influence cognition such as plasma cytokines and NFL levels, APOE genotype, comorbidities, and SARS-CoV-2 IgG antibody response. We report results that may differentiate individuals with nCoV from CoV and healthy controls.

## 2. Materials and Methods

### 2.1. Study Participants

Volunteers from the San Francisco Bay area with a documented history of SARS-CoV-2 infection as evidenced by a positive viral RNA PCR test result from nasal or throat swab were recruited into the LIINC observational cohort study through clinician referrals and participant self-referrals. All participants had recovered from acute illness due to SARS-CoV-2 virus infection, defined by resolution of fever >72 h, elapse of at least 21 days since illness onset, and overall improvement in COVID-19 symptoms. Exclusion criterion included a transfusion-dependent anemia or inability to provide informed consent. All volunteers signed a written informed consent approved by the University of California, San Francisco Institutional Review Board (IRB# 20-30479). Sixteen COVID-19 volunteers without neurological symptoms (CoV) and 8 COVID-19 volunteers with neurological symptoms (nCoV), which were the only available participants at the time of recruiting, were included in this analysis. During the first study visit, each participant underwent a detailed interview with a clinical research coordinator supervised by an infectious disease clinician (M.J.P.). The clinical research coordinator asked questions pertaining to disease symptoms; pre-existing comorbidities; and issues related to mobility, self-care, and ability to perform everyday activities. An itemized checklist was used to record each patient response at the worst point of the illness. The questions asked were adapted from the EQ-5D [30] with possible responses falling into 3 generalized levels scored numerically: 1 = no issues, 2 = some challenges, and 3 = severe difficulty (such as being bedbound or total lack of ability to engage in regular activities). We opted for a simple functional scale that could be easily monitored over time because COVID-19 patients experience a myriad of heterogeneous clinical presentations.

Whole blood was collected in EDTA tubes between April and May 2020. Plasma and PBMCs were collected and frozen in aliquots at −80 °C. The samples analyzed in this report were collected during the individuals’ first study visit, which ranged from 30 to 103 days post-symptom onset. At the time of recruiting, we were not able to differentiate recovered asymptomatic from uninfected individuals as SARS-CoV-2 antibody was found to be negative in some post-COVID-19 individuals (as shown in this study). Thus, we included 12 pre-pandemic healthy control plasma samples from a previous study that were frozen at −80 °C for comparative analysis. Of the 12 pre-pandemic controls, 6 were not available in sufficient quantities to be included in all assays.

### 2.2. SARS-CoV-2 IgG Antibody Test

IgG antibodies to SARS-CoV-2 nucleocapsid protein were determined qualitatively using a chemiluminescent microparticle immunoassay (CMIA) on the ARCHITECT i System from Abbott Laboratories (Abbott Park, IL, USA; catalog # 06R8620) according to manufacturer’s instructions. The sample/calibrator ratio (S/C), which linearly corresponds to IgG concentrations, was reported as an index. Index (S/C) was considered negative if <1.4 and positive if ≥1.4.

### 2.3. APOE4 Genotyping

The APOE gene is polymorphic at two single nucleotides, rs429358 and rs7412, resulting in 3 alleles, ε2 (T/T), ε3 (T/C), and ε4 (C/C), and their combinations resulting in 6 genotypes (ε2/ε2, ε/ε3, ε2/ε4, ε /ε3, ε3/ε4, and ε4/ε4). The worldwide frequencies of ε2, ε3, and ε4 alleles are 8.4%, 77.9%, and 13.7%, respectively. Frozen PBMCs were thawed in a 37 °C water bath. Cells were collected by centrifugation at 500× *g* for 10 min. DNA was isolated using the AllPrep DNA/RNA Mini kit (Qiagen, Germantown, MD, USA). DNA was stored at −20 °C until use. Taqman SNP Genotyping Assays for rs429358 and rs7412 (Thermo-Fisher) were used, and PBMC DNA was amplified using the ABI ViiA 7 instrument for endpoint PCR. Data were analyzed using the Taqman SDS software (Thermo-Fisher Scientific, Inc., Waltham, MA, USA). A positive control for ε3/ε4 was included in the assay (Coriell Institute for Medical Research, Camden, NJ, USA).

### 2.4. Plasma Multiplex Cytokine Analysis and NFL by MSD Assays

Seven plasma cytokines (IL-1β, IL-4, IL-6, IL-8, IL-10, TNFα, and IFNγ) and NFL protein were measured using chemiluminescence-based assays from Meso Scale Discovery (MSD, Gaithersburg, MD, USA). For the 7 cytokines, a V-PLEX Viral Panel 2 Human Kit was used (MSD, catalog # K15346D-1). For NFL, an R-PLEX Human Neurofilament L Kit was used (MSD, catalog # K1517XR-2). The detection ranges are IFNγ 0.366–1500 pg/mL, IL1β 0.149–610 pg/mL, IL4 0.0515–211 pg/mL, IL6 0.168–690 pg/mL, IL8 0.149–612 pg/mL, IL10 0.0869–356 pg/mL, TNFα 0.0896–367 pg/mL, and NFL 5.5–50,000 pg/mL. All assays were performed in duplicate. Analyses were done using a QuickPlex SQ 120 instrument (MSD) and DISCOVERY WORKBENCH^®^ 4.0 software. All samples were run at the same time.

### 2.5. nEV Isolation

All nEV samples, COVID-19 and controls, were isolated by the same operator at the same time from frozen plasma as previously described [20]. Briefly, plasma fibril and coagulation proteins were removed by adding 1.25 units of thrombin to 250 μL of plasma for 1 hr, followed by centrifugation at 3000× *g* for 20 min. Total EVs were then precipitated by adding 126 μL of ExoQuick^TM^ Exosome Precipitation Solution (Systems Biosciences, Palo Alto, CA, USA; catalog # EXOQ20A-1) to the clarified plasma in the presence of protease and phosphatase inhibitors. The precipitated total EV pellets were resuspended and incubated with biotinylated L1CAM monoclonal antibody (Thermo-Fisher Scientific; catalog # 13-1719-82), a protein on the surface of neurons throughout the nervous system, followed by capture of labeled EVs with streptavidin-conjugated agarose beads (Pierce^TM^ Streptavidin Plus UltraLink^TM^ Resin from Thermo-Fisher Scientific; catalog # 53117). nEV-resin complexes were washed and the nEVs released from the beads using a 100 μL solution of 50 mM Glycine-HCl (pH3). Released nEVs were neutralized with 10 μL 1M Tris-HCl (pH8) and stored at −80 °C or lysed with 390 μL lysis buffer containing a final concentration of 0.15% BSA, 1 X protease, phosphatase inhibitors, and M-PER^TM^ Mammalian Protein Extraction Reagent (Thermo-Fisher Scientific; catalog # 78501) and then stored at −80 °C until use.

### 2.6. Characterization of nEVs by NTA and electron microscopy 

Nanoparticle tracking analysis (NTA) was performed on the nEV samples to determine size and vesicle number. Data were generated using a NanoSight LM10 instrument (Malvern Instruments, Malvern, United Kingdom) with a 405 nm laser-equipped sample chamber as previously described [17]. Results were analyzed using NTA 3.3 software. Each sample analysis consisted of three 40 s video recordings. Mode particle sizes were reported due to the skewed distributions.

Transmission electron microscopy (TEM) was performed on a subset of the nEVs isolated from patient plasma. In brief, eluted nEVs were fixed in 4% buffered paraformaldehyde (PFA) and deposited onto Formvar carbon-coated electron microscopy nickel grids for 5 min. The excess fluid was blotted off with #1 filter paper, and the grids were stained with saturated uranyl acetate solution (Ted Pella, Inc., Redding, CA, USA) for 5 s. Excess fluid was then blotted off again, and the grids dried overnight. Visualization of EVs was performed using a Technai 10 transmission electron microscope (Field Electron and Ion Co. Hillsboro, OR, USA).

### 2.7. Neuronal-Enriched EV Protein Cargo Analyses 

Luminex bead assays were performed on the nEV lysates using a Neurodegeneration 9-plex Human ProcartaPlex^TM^ panel from ThermoFisher (catalog #EPX090-15836-901) with a Luminex LX200 instrument using Luminex xMAP Technology. The procedure was performed according to the manufacturer’s instructions. Results were analyzed using Milliplex^TM^ Analyst version 5.1 software. The 9 nEV proteins analyzed by the Luminex assay were Aβ1-40 (detection range: 451–1,847,000 pg/mL), Aβ1-42 (range: 0.42–1700 pg/mL), fibroblast growth factor 21 (FGF21) (range: 8.64–35,400 pg/mL), kallikrein-related peptidase 6 (KLK6) (range: 5.57–22,800 pg/mL), Neural Cell Adhesion Molecule 1 (NCAM1) (range: 54–221,900 pg/mL), Neurogranin (NRGN) (range: 10–41,600 pg/mL), TAR DNA Binding Protein (TDPBP) (range: 96–393,200 pg/mL), total Tau (range: 20–80,000 pg/mL), and p-T181-tau (range: 1.95–2000 pg/mL).

The MSD assay for NFL protein was performed on the nEV lysates using an R-PLEX Human Neurofilament L Kit (MSD, catalog #K1517XR-2) according to manufacturer’s instructions with the exception of the use of undiluted samples. The detection range for NFL is 5.5–50,000 pg/mL. Analyses were done using a QuickPlex SQ 120 instrument (MSD) and DISCOVERY WORKBENCH^®^ 4.0 software. 

ELISA was performed on the nEV lysates to determine protein concentrations for CD81, Programmed Cell Death 6 Interacting Protein (PDCD6IP, also known as ALIX), Synaptophysin (SYP), and High Mobility Group Box 1 (HMGB1) using commercially-available kits as follows: CD81 (American Research Products, Inc., Waltham, MA, USA; catalog # CSB-EL004960HU, detection range: 0.156–10 ng/mL), ALIX (Lifeome, Oceanside, CA, USA; catalog #CSB-EL017673HU, range: 47–3000 pg/mL), SYP (Novus Biologicals, Centennial, CO, USA; catalog #NBP2-80283, range: 0.031–2 ng/mL), and HMGB1 (Novus Biologicals; catalog #NBP2-62766, range: 0.031–2 ng/mL). All ELISAs were performed according to manufacturer’s instructions, and each sample was analyzed in duplicate. Protein concentrations were determined by absorbance using a Spectra Max M5 plate reader (Molecular Devices) with Softmax Pro 5 software.

### 2.8. Statistics and Bioinformatics

As an exploratory study at the beginning of the pandemic, we took the first 16 individuals entered into the LIINC cohort with lingering COVID-19 symptoms and 8 individuals with continued neurological complaints. We used plasma collected from 12 healthy controls, 6–12 months pre-pandemic. Nonparametric Wilcoxon test was used to compare the means of two groups. Multiple group means were compared with the nonparametric Kruskal–Wallis test followed by post hoc Dunn test with Benjamini and Hochberg multiple comparison (BH) corrections. Spearman’s correlations were used for correlation analysis. Receiver operator curve (ROC) analyses were performed for plasma cytokines. To evaluate variances of EV particle sizes, the particle sizes were reconstructed from the NTA summary data acquired from the NanoSight instrument, which generated hundreds of millions of data points. We randomly sampled 10^5^ data points for each sample in order to be handled by a desktop computer. Variance of the sizes among the groups were compared with a Levene test. All statistical analyses were performed in R version 4.0.2. Selected proteins were analyzed for functional or Gene ontology (GO) enrichment using DAVID [31], GOnet [32] and Cytoscape [33] tools. Ingenuity Pathway Analysis software (Qiagen) [34] was used for enrichment and pathway analysis. 

## 3. Results

### 3.1. Participant Demographic and Clinical Data

Twenty-four participants with COVID-19 as confirmed by SARS-CoV-2 nucleic acid testing of respiratory samples in the LIINC study were included in this analysis. Of these 24, eight had self-reported neurological symptoms and 16 did not. In addition to the 24 LIINC participants, we also included 12 pre-COVID-19 historical controls. The majority of the study participants were women (22 of 36). The mean ages of the COVID-19 participants (45.3 ± 12.7) were similar to that of the control participants (52.3 ± 12.4, *P* = 0.316) (Table 1). In the control group, 33.3% were women, while in all COVID-19 participants, 75% were women (Table 1). Two of the eight with neurological signs had an APOE ε3, ε4 genotype, and five of the 16 CoV participants had an APOE ε3, ε4 or ε4, ε4 genotype (Table 1). Of the 16 CoV participants, three had been hospitalized, while half of the nCoV participants were hospitalized (Table 2). The number of days from the onset of symptoms to the first in-person research visit (days till visit) varied from 30 to 103 days (median 60 days, interquartile range (IQR): 40.75–85.0 days). There was no significant difference between CoV (56.6 ± 20.3) and nCoV (76.5 ± 27.2) for days till visit (Student’s t test *P* = 0.094). In the cases described, neurological symptoms persisted beyond the infectious period and remained present at the time of the first visit, but other symptoms (fever, cough, etc.) resolved. Twenty-one of the 24 participants reported some mobility, selfcare, or activity issues during their acute illness (Table 2) [12]. Eight reported neurological symptoms that were primarily related to memory and cognition, with one reporting double vision and one reporting hallucinations (Table 2). None of the COVID-19 participants had a history of immunosuppression, cancer, heart, or kidney disease. One had HIV, four had diabetes, five had hypertension, and five had lung disease (Table 2). All participants with nCoV had at least one comorbid condition, while only seven of 16 CoV participants (43.8%, *P* = 0.0095) had any comorbid condition. Lung disease was defined as lung problems that have persisted in the last 5 years, such as asthma, chronic obstructive pulmonary disease, emphysema, or bronchitis. Autoimmune disease was defined as any autoimmune disease, such as rheumatoid arthritis, lupus, Crohn’s disease, or ulcerative colitis.

### 3.2. Plasma Cytokines, NFL, and IgG Levels

**Figure 1:**
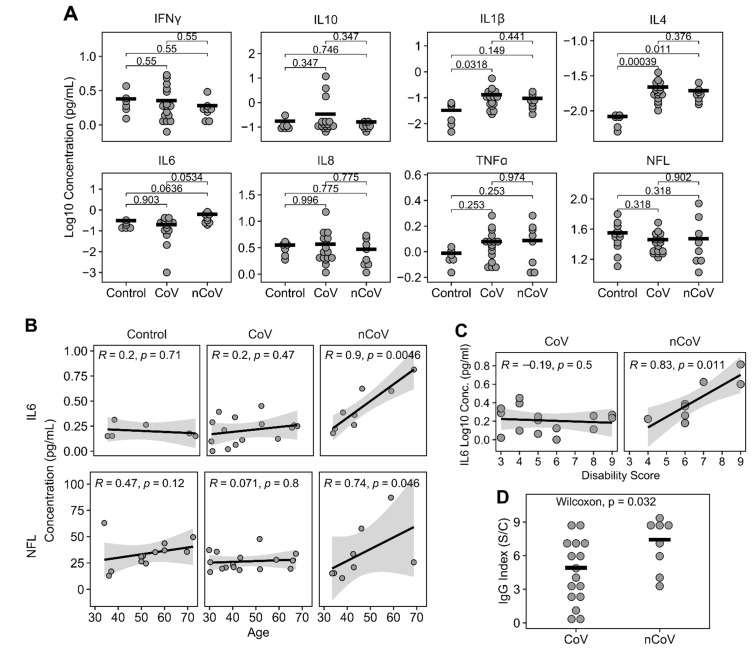
Plasma cytokines and NFL levels. (**A**) Meso Scale Discovery (MSD) analysis for 7 cytokines and NFL showed a significant increase of IL4 in nCoV and CoV compared to Controls. IL6 was trending up in nCoV compared to CoV and Controls. Post hoc Dunn tests with BH correction were used for comparisons of group means and shown in the figure. *n* = 6 in all assays except NFL (*n* = 12) for Control, *n* = 16 for CoV, and *n* = 8 for nCoV. (**B**) IL6 and NFL strongly correlated with increasing age in nCoV but not in Control or CoV groups. Spearman’s correlations were used. Shaded areas indicate 95% confidence interval. Control (*n* = 6 for IL6, *n* = 12 for NFL), CoV (*n* = 15 for IL6 and *n* = 16 for NFL), and nCoV (*n* = 8 for IL6 and NFL). (**C**) IL6 and Disability Score strongly correlated in nCoV (*n* = 8), but not in CoV (*n* = 16). (**D**) Levels of IgG showed differences between CoV (*n* = 16) and nCoV (*n* = 8) participants. Horizontal bars indicate group means. Each circle is an individual. Horizontal bars indicate means of the groups.

To determine whether participants experienced ongoing elevations in levels of peripheral markers of inflammation, a seven-multiplex MSD cytokine array plus a single-plex MSD NFL assay were performed on plasma (Figure 1A). There were no differences between IFNγ, IL-10, IL-8, TNFα, or NFL levels in samples from CoV and nCoV participants compared to control samples using the non-parametric Kruskal–Wallis analysis of variance tests. However, IL1β (*P* = 0.038, *n* = 30), IL4 (*P* = 0.00064, *n* = 30) and IL6 (*P* = 0.0397, *n* = 30) showed significant differences among the groups by Kruskal–Wallis tests. Post hoc Dunn tests with Benjamini and Hochberg multiple comparison correction were subsequently used to further elucidate differences between each pair of the groups. IL-1β was significantly increased in the CoV group (log10 median −0.98 pg/mL, IQR −1.28–−0.78, *n* = 16) compared to controls (log10 median −1.58 pg/mL, IQR −2.0–−1.22, *n* = 6; *P* = 0.032). IL-4 was significantly increased in both the CoV (log10 median −1.72 pg/mL, IQR −1.79–−1.64; *P* < 0.001, *n* = 16) and nCoV (log10 median −1.77 pg/mL, IQR −1.84–−1.72; *P* = 0.011, *n* = 8) groups compared to controls (log10 median −2.1 pg/mL, IQR −2.2–−2.1, *n* = 6). IL-6 was trending up only in the nCoV group (log10 median −0.43 pg/mL, IQR −0.60–−0.22, *n* = 8; *P* = 0.053) compared to CoV (log10 median −0.63 pg/mL, IQR −0.98–−0.55, *n* = 15) and controls (log10 median −0.78 pg/mL, IQR −0.82–−0.62; *P* = 0.064, *n* = 6). ROC analyses showed IL1β, IL4, and IL6 had predictive values for various groups as shown in Supplement Figure S1. IL1β showed a significant AUC (0.833, *P* = 0.017) for CoV compared to controls. IL4 showed significant AUCs in both nCoV (1, *P* = 0.001) and CoV (1, *P* < 0.0001) compared to controls. IL6 showed a significant AUC for nCoV compared to controls (0.875, *P* = 0.02) and a trending significant AUC for nCoV compared to CoV (0.734, *P* = 0.07). We used Spearman correlation to determine if a specific cytokine or NFL would be associated with age in each of the three groups. The only significant correlations were with IL-6 (Spearman *R* = 0.9, *P* = 0.0046, *n* = 8) and NFL (Spearman *R* = 0.74, *P* = 0.046, *n* = 8), which correlated with age in the nCoV group but not the control or CoV groups (Figure 1B). Because plasma cytokines and, in particular, IL-6 have been associated with intellectual and age-related disability [35,36,37], we looked for any correlations with the plasma analytes and disability as determined by a numerical score for mobility, selfcare, and activity issues. Only IL-6 had a significant correlation with everyday activities (Spearman’s correlations, *R* = 0.83, *P* = 0.011) (Figure 1C). IgG levels were significantly higher in nCoV (median 7.90 index S/C, IQR 5.5–8.7, *n* = 8) compared to CoV participants (median 4.38 index S/C, IQR 2.5–6.8, *n* = 16, Wilcoxon *W* = 29, *P* = 0.032) (Figure 1D). Surprisingly, three CoV participants had negative IgG levels (<1.4; Table 2). IgG levels did not correlate with days till visit (Spearman correlation *R* = 0.28, *P* = 0.185, *n* = 24).

### 3.3. Neuronal Enriched EV Characterization

**Figure 2:**
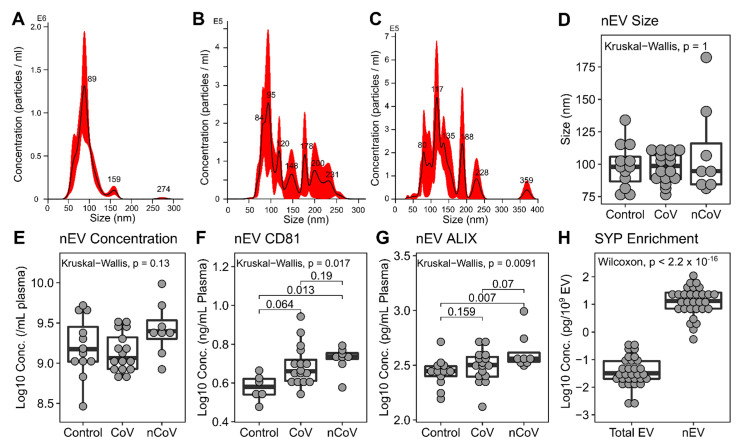
nEV characterization. Representative NTA spectra from Control (**A**), CoV (**B**), and nCoV (**C**) showing the heterogeneous nature of the nEVs from COVID-19 participants. Variance of sizes were significantly different among the groups, Levene test *p* < 0.0001. nEV sizes determined by NTA (**D**) for Control (*n* = 12), CoV (*n* = 16), and nCoV (*n* = 8) showed no difference between the groups. nEV concentrations were determined by NTA (**E**) (*n* is the same as panel D). nEV protein cargo for CD81 (**F**), ALIX (**G**), and SYP (**H**) were determined using ELISA. CD81 showed an increase in nCoV (*n* = 8) compared to Control (*n* = 6). ALIX increased in nCoV (*n* = 8) compared to Control (*n* = 12) but not significantly to CoV (*n* = 16). (H) Synaptophysin (SYP) is enriched in nEV (*n* = 30) compared to total EVs (all EVs in plasma, *n* = 30). Kruskal–Wallis test was used and followed with post hoc Dunn tests if significant for groupwise differences.

nEVs were isolated from the plasma using immunoadsorption. We characterized nEVs by nanoparticle tracking analysis (NTA) for size and concentration. nEVs from control samples were typically 75–125 nm and were fairly consistent in size (Figure 2A). Electron microscopy confirmed their cup-shaped morphology, typical of vesicles with homogeneously sized EVs (Supplementary Figure S2A). In contrast, nEVs from CoV participants without neurological symptoms were more diverse in size (Levene test *F* = 2397.3, *P* < 0.0001, Figure 2B) and had a similar NTA profile to nCoV nEVs (Figure 2C). However, the overall size and concentrations were not different among the three groups (Figure 2D,E). Electron microscopy showed a diverse size range in the EVs from nCoV participants (supplementary Figure S2B). EV protein markers CD81 and ALIX were both elevated in the nCoV nEVs above the Controls (Figure 2F,G). As EV concentrations were consistent across the groups, they were used for EV cargo protein normalization in subsequent experiments. Synaptophysin (SYP) was enriched in all nEVs compared to all EVs in plasma (total EVs), strongly suggesting a neuronal origin (Figure 2H). 

### 3.4. Neuronal enriched EV protein cargo

**Figure 3:**
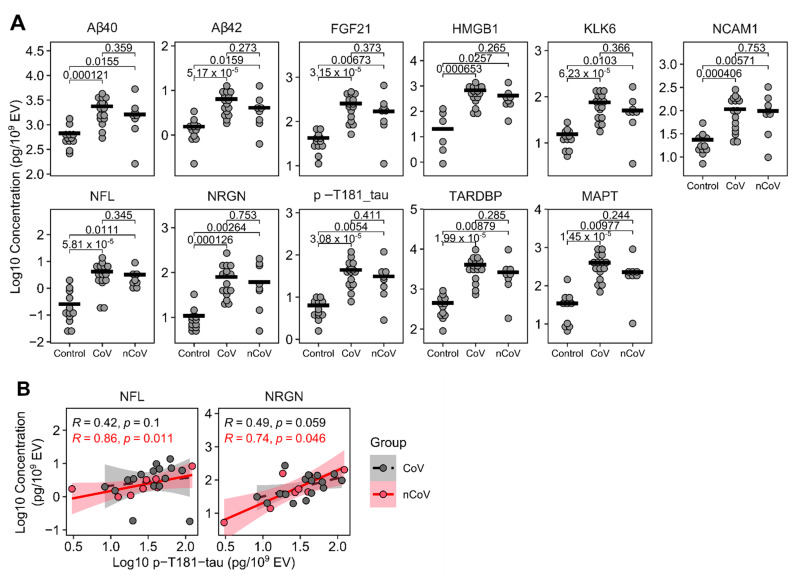
Protein levels of nEV cargo. (**A**) nEV lysates were analyzed using MSD (NFL), ELISA (HMGB1), and Luminex (the rest). Concentrations were normalized to nEV counts. All nEV proteins showed significant increase in both CoV (*n* = 16) and nCoV (*n* = 8) participants compared to historic Control participants (n = 12 except HMGB1 where *n* = 6). No differences were found between the CoV and nCoV groups. Kruskal–Wallis tests showed groups were different in all proteins (*p* < 0.01 for all proteins). Subsequent post hoc Dunn tests with BH correction were performed for groupwise comparisons and are shown in the figures. Horizontal bars indicate group means. (**B**) nEV p-T181-tau significantly correlated with NFL or neurogranin (NRGN) in nCoV (*n* = 8), but not in the CoV group (*n* = 16) (Spearman’s correlation).

**Figure 4:**
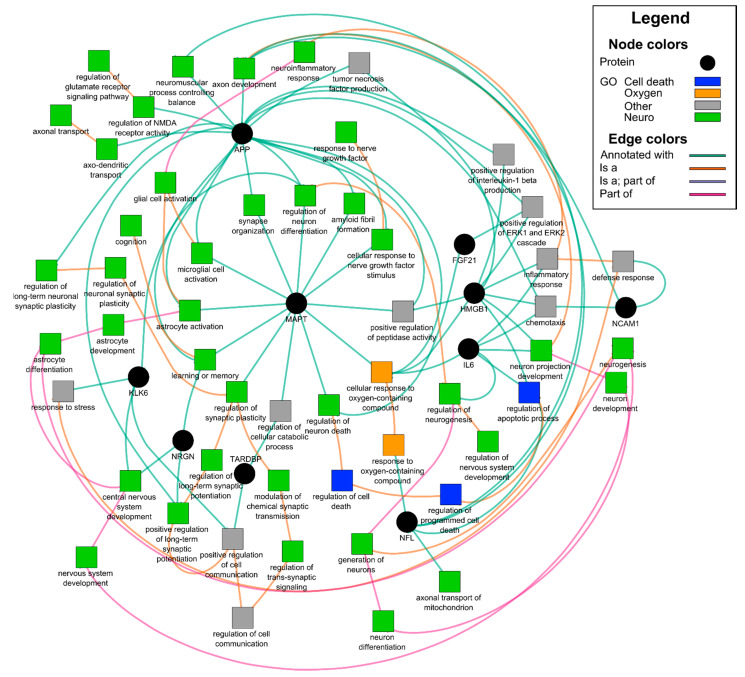
Gene ontology network of 10 selected proteins. Proteins were analyzed using GOnet and visualized using Cytoscape. Interested GO items are colored, non-relevant GO items were removed for clarity. Black circles are proteins analyzed and squares are related GO items. Nervous system-related GO terms are colored green, cell death-related colored blue, oxygen-containing compound-related items colored orange, and others are colored gray.

EVs carry diverse cargo that can be a reflection of the parent cell, in this case neurons, and a mechanism for eliminating toxic waste. We lysed the nEVs and looked at proteins associated with neural damage using a 9-plex neurodegeneration assay (Luminex). Amyloid beta (Aβ) 1-40, Aβ1-42, FGF21, HMGB1, KLK6, NCAM1, NFL, NRGN, p-T181-tau, SYP, TARDBP, and MAPT (total tau) (Figure 3A) were normalized to nEV counts. Surprisingly, all the proteins tested were significantly elevated using the nonparametric Kruskal–Wallis tests followed by Dunn post hoc tests with BH correction in both the CoV (N = 16) and nCoV (N = 8) groups compared to healthy controls (N = 6 or 12), strongly suggesting they are all interrelated (Figure 3A). HMGB1 is a ubiquitous nuclear protein that, when released extracellularly, promotes inflammation and cytokine release [38]. Importantly, this release within the brain can activate microglia, and when released via EVs into the periphery, can activate monocytes to promote further inflammation. We previously reported that nEV HMGB1 and NFL were elevated in HIV-infected individuals with cognitive impairment [20]. To explore any relationships between the nEV target proteins, we performed Spearman rank correlation analyses. NFL and neurogranin (NRGN), a postsynaptic protein, were both elevated in the CoV and nCoV groups compared to the controls (Figure 3A). There were significant correlations of both NFL (Spearman *R* = 0.86, *P* = 0.011, *n* = 8) and NRGN (Spearman *R* = 0.74, *P* = 0.046, *n* = 8) with p-T181-tau in the nCoV participants but not in CoV (Figure 3B). The elevation of the other neural proteins is not entirely surprising, as they are interrelated in a neuronal network (Table 3 and Figure 4). Gene ontology network analysis showed that the target proteins (black circles in Figure 4) are related to a few common and important gene ontology categories, such as synaptic functions, glial cell activation, and neuron stress or death pathways. Using functional annotation analysis from DAVID and Ingenuity Pathway Analysis, a few of the proteins upregulated in nEVs after SARS-CoV-2 infection showed enrichment in neuron death, neurodegeneration, and synaptic plasticity (Table 3). They are also involved in genetic associated diseases such as dementia and schizophrenia. A few common transcription factors were also associated with these proteins, such as NF-κB and STAT3 (Table 3). A number of the other targets tested (Figure 3A), KLK6, a protein that promotes inflammation [39]; TARDBP (TDP-43), a protein that promotes p53-mediated neuronal death [40]; and FGF21, which reacts to stress-sensing pathways [41], are all involved with neuron and cell death pathways. Likewise, amyloid precursor protein (APP), which is abundant in neurons, and IL-6 are predominant in all the pathways except synaptic plasticity (Table 3). These proteins are involved in different states of neural system functions such as synaptic plasticity, astrocyte and microglia activation, neural cell death, or apoptosis, suggesting they may all play a role in the COVID-19-associated neurological manifestations (Figure 4). 

## 4. Discussion

As the COVID-19 pandemic continues, there is growing concern that a substantial proportion of individuals infected with SARS-CoV-2 may develop long-term sequelae, including various neurocognitive and other neurological symptoms that could have a major impact on return to everyday activities and quality of life. The aim of this study was to see if those individuals with cognitive dysfunction or neurological complaints exhibited a common underlying mechanism. 

Elevated cytokine secretion has been reported in both acute and mild cases of COVID-19 and has been used to follow disease progression [42]. Continued inflammation for select cytokines tested in this study was apparent in the 24 COVID-19 participants. IL-4 continued to be significantly elevated in all COVID-19 participants. IL-4 is a cytokine involved in brain function such as memory, and its role is beneficial and counteractive to proinflammatory cytokines [43]. Its elevation may signal a response to the neuroinflammation threat and attempt to restore homeostasis. IL-6 was not significantly increased, but trended higher in the nCoV group compared to CoV and the control group. A recent report showed that IL-6 and IL-10 predicted COVID-19 severity confirmed by a ROC analysis (AUC = 0.841 and 0.822, respectively) [44,45,46]. While we did not see an increase in IL-10 in the CoV and nCoV groups, IL-6 levels were trending up in the participants experiencing neurological symptoms. Fifty percent of the nCoV participants were hospitalized compared to 19% (3/16) of the CoV group. Only one patient was intubated out of the 24 COVID-19 patients studied. IL-6 is a pleiotropic cytokine that is associated with the acute phase of inflammation, and when persistent with aging, can predict disability [37]. This was a fairly young group of COVID-19-infected participants with a mean age of 45 years. We looked at all the cytokines tested plus NFL and any correlation with age in the CoV and nCoV groups. IL-6 and NFL correlated with increasing age only in the group of nCoV participants. Increased plasma NFL has been associated with ongoing CNS injury in HIV [47], Parkinsons [48], and AD [49]. Anti-SARS-CoV-2 IgG levels were also significantly elevated in the nCoV group compared to the CoV group. This may reflect more severe illness, and it did not signify further time out from infection. Additional studies in larger longitudinal cohorts are warranted to determine resolution of these indices. 

ApoE4, a genetic risk factor for Alzheimer’s, increases the risk for severe COVID-19 infection [50]. In one UK study, APOE ε4, ε4 accelerated the risk of severe COVID-19, independent of pre-existing dementia, cardiovascular disease, and type 2 diabetes [51]. The apolipoprotein ε4 isoform with two copies of ε4 has also been a factor in late-onset AD contributing to Aβ accumulation, increased neurofibrillary tangles, and increased neuroinflammation. While our numbers are small, we did not see a difference in this genotype between groups, although this is a relatively young cohort, and the effects of this genotype would not manifest until later or be more obvious in older patients with COVID-19.

While CSF is thought to be the best reflection of brain health, it is an invasive procedure, and like plasma, contains a complex protein profile. Neuronal EVs may better reflect the ongoing health of the neurons and are fairly easy to isolate and interrogate cargo. Neuronal EVs can promote neurogenesis [52] and shuttle normal signaling within the CNS. They can also remove excess or damaged proteins, spread toxic Aβ, and hyperphosphorylated tau between cells and activate other neural cells [22,53,54]. CSF biomarkers of neurodegeneration include total tau, phosphorylated tau, Aβ42, and NFL, which are all associated with AD [55]. In this report, we showed that all of these protein biomarkers were elevated in nEVs. Numerous nEV studies on AD parallel these CSF findings, suggesting that the less invasive nEVs may be a good alternative to CSF [22,26]. EVs have been used as predictive biomarkers of cognitive impairment before frank dementia [22]. In a recent large longitudinal study to predict AD, increased nEV diameter and cargo of several phosphorylated taus, including p-T181-tau and insulin signaling molecules, were able to predict AD with excellent accuracy [27]. 

Other neurological studies using plasma nEVs showed that total tau, Aβ42, p-T181-tau, and IL-6 were all significantly increased in both acute and chronic TBI [56]. Mild TBI also showed an increase in nEV Aβ42 and neurogranin [25]. Total tau was significantly increased in nEVs from Parkinson’s disease (PD) over AD or control nEVs [57]. We reported that Aβ42, HMGB1, and NFL were increased in nEVs of cognitively impaired individuals with HIV [20,21]. While COVID-19, HIV, TBI, AD, and PD all have similar nEV cargo and inflammation as a common thread, they have distinctly different but often overlapping nEV patterns. The literature predicts that elevated Aβ42, NFL, and phosphorylated tau in nEVs over time and aging contribute to neurodegeneration that may end with AD [22]. Surprisingly, nEV cargo-containing proteins associated with stress, memory formation, and neurodegeneration were elevated in all participants with COVID-19 versus the control group. 

There are a number of limitations of this study. The sample size included in the analysis was small and not reflective of the larger epidemic in the San Francisco Bay Area. Control and COVID-19 participants were not well matched by sex. COVID-19 participants gave self-reported symptoms with no objective measure of neurological function. These participants were among the first self-reports of neurological sequelae from post-COVID-19 cases reported. The nCoV participants had more co-morbidities that may explain the neurological complaints. Detailed neuropsychological testing and MRI were not performed for this study due to limitation of personnel and resources in the early pandemic environment and are part of future analyses. Timing of plasma collection also differed between the control and CoV participants. Previous studies using frozen plasma/serum to isolate nEVs from HIV-infected individuals and individuals diagnosed with AD utilized biobanks and also had disparate collection timing [21,27]. 

We were surprised to find that the cargo of the nEVs from all recovering COVID-19 participants, regardless of time after infection, was altered, as they all showed increased inflammatory and neurodegenerative proteins. As studies looking at human nEV cargo have not been performed to our knowledge on other virus infections with the exception of HIV [20,21,28], we do not know if the neurodegenerative proteins present in the nEVs of COVID-19 recovered individuals are transient or long-term. If transient, it may reflect ongoing neuroinflammation and a healthy continued elimination of toxic proteins from neurons and subsequent removal *in situ* by microglia or by circulating peripheral scavenger cells. Alternatively, if long-term, the condition is worrisome, as it may signal continued neuroinflammation or a possible precursor to neurodegeneration, as the cargo contains inflammatory promoters. The Gene Ontology analysis suggests there may be synaptic disruption and possibly neuronal damage. More individuals recovering from COVID-19, especially older survivors, need to be studied longitudinally to determine if these results are persistent, if neurological complaints continue in a subset of COVID-19 survivors, and whether individuals without overt neurological complaints are truly not affected long-term. 

In summary, this study suggests that recovery from SARS-CoV-2 infection in some individuals may have lingering neurological effects, both perceived and occult. Elevated antibody response, a trending increase in plasma IL-6 levels, and comorbidities were all associated with neurological manifestations in COVID-19. Additionally, nEV cargo containing neurodegenerative proteins are being shuttled from neurons into the periphery of all post-COVID individuals studied. Further studies are needed to determine whether these nEV toxic proteins diminish with time. 

## Figures and Tables

**Table 1:** Participant demographics.

		Control (%) ^†^		CoV (%)		nCoV (%)		*p*-Value
**Sex**	Female (N = 22)	4	(33.3)	12	(75.0)	6	(75.0)	0.053
	Male (N = 14)	8	(66.7)	4	(25.0)	2	(25.0)	
**Race**	Asian	2	(16.7)	0	(0.0)	1	(12.5)	0.154
	Black	4	(33.3)	2	(12.5)	1	(12.5)	
	White	6	(50.0)	11	(68.8)	5	(62.5)	
	American Indian	0	(0.0)	0	(0.0)	1	(12.5)	
	Undisclosed	0	(0.0)	3	(18.8)	0	(0.0)	
**Ethnicity**	Hispanic	1	(8.3)	3	(18.8)	4	(50.0)	0.081
	Non-Hispanic	11	(91.7)	13	(81.3)	4	(50.0)	
**APOE genotype**	ε2, ε3	NA	NA	2	(12.5)	0	(0.0)	0.615
	ε3, ε3	NA	NA	9	(56.3)	6	(75.0)	
	ε3, ε4	NA	NA	4	(25.0)	2	(25.0)	
	ε4, ε4	NA	NA	1	(6.3)	0	(0.0)	
								
**Comorbidity**		NA	NA	7	(43.8)	8	(100)	0.0095
								
**Days till visit, Mean (SD)**		NA		56.6 (20.3)		76.5 (27.2)		0.094
								
**Age in years, Mean (SD)**		52.3 (12.4)		45.2 (13.2)		45.6 (12.3)		0.316

^†^ Controls are COVID-19 negative historic samples. Sex, race, ethnicity, APOE genotype, and comorbidity were represented as counts and percentages in each group and tested with Chi-squared tests or Fisher’s exact test. Age was represented as mean (SD) and tested with ANOVA. Days until visit was defined as days of symptoms before first visit, represented as mean (SD) and tested with Student’s t test.

**Table 2:** Clinical features of the 24 COVID-19 patients.

ID	Age	Sex	Race/Ethnicity	Mobility Issues ^‡^	Selfcare Issues ^‡^	Activity Issues ^‡^	Hospitalized	Days Till visit ^§^	IgG	ApoE Genotype	Neuro Symptoms	Comorbidity
1	34	F	AI/L	2	1	1	No	36	4.2	ε3,ε3	DV	LD
2 ^†^	69	M	W	3	3	3	No	62	8.3	ε3,ε3	M/C	HT,LD,O
3	38	F	Asian	2	2	2	Yes	103	8.6	ε3,ε3	H	AID, DM,O
4 ^†^	59	M	W	3	3	3	Yes	80	7.5	ε3,ε3	M/C	HT,LD
5	33	F	W/L	2	1	3	No	97	3.3	ε3,ε3	M/C	HT
6	43	F	B/L	2	1	3	Yes	99	9.4	ε3,ε4	M/C	HT,LD,O
7	46	F	W/L	2	2	3	Yes	95	5.9	ε3,ε4	M/C	DM,O
8	43	F	W	2	2	2	No	40	9.1	ε3,ε3	M/C	O
9	30	F	W	1	2	3	No	38	2	ε2,ε3	None	
10	37	F	W	1	1	1	No	30	0.4	ε3,ε3	None	
11	30	F	W	1	1	1	No	31	3.9	ε3,ε3	None	
12	43	F	W	3	2	3	No	52	1.1	ε3,ε4	None	
13	35	F	W	2	1	2	No	41	8.8	ε3,ε4	None	
14	30	F	W	2	1	1	No	44	6.1	ε3,ε4	None	
15	66	F	W	3	2	3	No	49	8.4	ε3,ε3	None	LD
16	40	F	B	1	1	1	No	38	4.9	ε3,ε4	None	O
17	33	F	W	1	2	1	No	60	3.5	ε3,ε3	None	O
18	67	M	W	2	1	2	No	64	2.6	ε4,ε4	None	O
19	43	F	B	1	3	2	No	70	7.3	ε3,ε3	None	HT
20	59	F	W	2	2	2	No	60	0.3	ε2,ε3	None	
21	40	F	W	2	1	2	No	60	7.2	ε3,ε3	None	
22	52	M	Dc/L	3	3	3	Yes	88	5.8	ε3,ε3	None	DM
23	65	M	Dc/L	3	3	3	Yes	84	6.7	ε3,ε3	None	DM,O
24	52	M	Dc/L	2	1	1	Yes	97	4.2	ε3,ε3	None	

^†^ Patient 2 was HIV +; all others were HIV-. Patient 4 was intubated; all others did not require intubation. ^‡^ 1 = none, 2 = some, 3 = bedbound or unable. **^§^** Days till visit was defined as days of symptoms before first visit. IgG levels of SARS-CoV-2 IgG < 1.4 were considered negative. Abbreviations used for race/ethnicity, W: White; B: Black; AI: American Indian; L: Latino; Dc = Declined; for neuro-symptoms, DV = Double vision, M/C = Memory/cognition, H = Hallucinations; for comorbidity, LD = Lung Disease, HT = Hypertension, AID = Autoimmune disease, DM = Diabetes, O = Obesity (BMI ≥ 30.0).

**Table 3:** DAVID functional annotation for nEV cargo from all COVID-19 volunteers.

Term	*p* Value	Genes
**Gene Ontology**		
GO:0030182~neuron differentiation	0.0002	APP, IL6, NCAM1, MAPT, HMGB1, KLK6
GO:0050803~regulation of synapse structure or activity	0.0002	APP, NCAM1, SYP, NRGN
GO:0007399~nervous system development	0.0003	APP, IL6, NCAM1, MAPT, HMGB1, NRGN, KLK6
GO:0070997~neuron death	0.0003	APP, IL6, FGF21, KLK6
GO:0022008~neurogenesis	0.0004	APP, IL6, NCAM1, MAPT, HMGB1, KLK6
GO:0031175~neuron projection development	0.0006	APP, IL6, NCAM1, MAPT, HMGB1
GO:0008219~cell death	0.0018	APP, IL6, HMGB1, TARDBP, FGF21, KLK6
GO:0048167~regulation of synaptic plasticity	0.0024	NCAM1, SYP, NRGN
GO:0006935~chemotaxis	0.0026	APP, IL6, NCAM1, HMGB1
GO:0030424~axon	0.0050	APP, MAPT, NRGN
Uniprotein: Neurodegeneration	0.0068	APP, MAPT, TARDBP
GO:0006915~apoptotic process	0.0096	APP, IL6, HMGB1, TARDBP, FGF21
GO:0048812~neuron projection morphogenesis	0.0323	APP, NCAM1, MAPT
GO:0099536~synaptic signaling	0.0393	NCAM1, SYP, NRGN
		
**Genetic Association Database**		
Dementia	0.0009	APP, IL6, MAPT
Alzheimer’s Disease	0.0013	APP, IL6, MAPT, TARDBP
Schizophrenia	0.0141	IL6, NCAM1, MAPT, NRGN
Neuroinflammation Signaling Pathway ^†^	0.0000	APP, IL6, HMGB1, MAPT
Neuroprotective Role of THOP1 in Alzheimer’s Disease ^†^	0.0000	APP, KLK6, MAPT
		
**Transcription factor binding site**		
NF_K_B	0.0298	APP, IL6, NCAM1, SYP, MAPT, TARDBP, NRGN, KLK6
STAT3	0.0217	APP, NCAM1, SYP, MAPT, TARDBP, FGF21, NRGN, KLK6

^†^ IPA analyses.

## Data Availability

All data is contained within the manuscript and supplementary materials.

## Supplementary Materials

The following are available online at https://www.mdpi.com/2073-4409/10/2/386/s1, Figure S1:Receiver-Operator curves for significantly differentially expressed plasma cytokines., Figure S2. Transmission electronic microscopy of nEVs from plasma.
