# Supplementary material for: Characterization and Biomarker Analyses of Post-COVID-19 Complications and Neurological Manifestations
Source: Cells. 2021 Feb 13;10(2):386. doi: 10.3390/cells10020386 (PMC7918597; doi:10.3390/cells10020386)
Supplement: Supplementary file 1 [file cells-10-00386-s001.pdf]

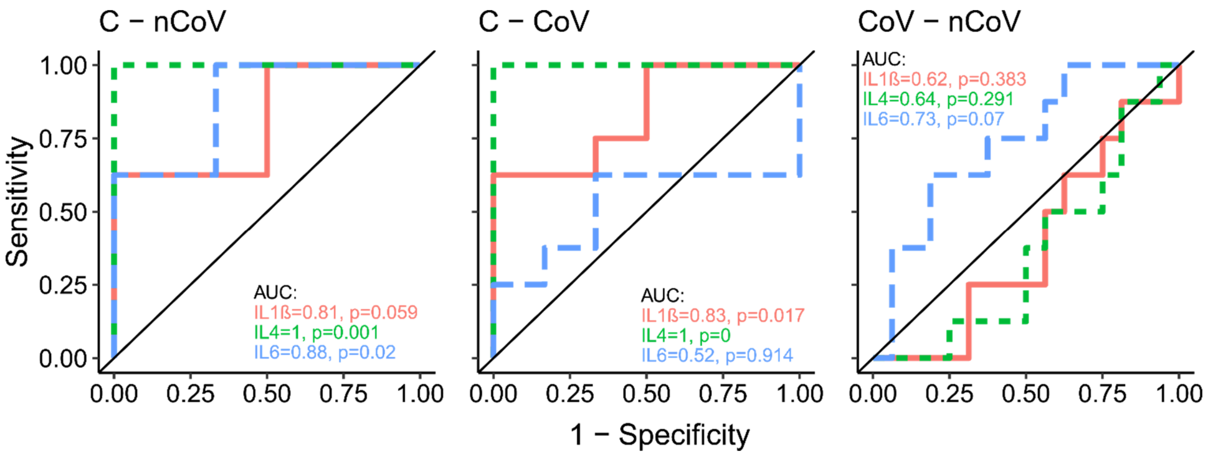

Figure S1. Receiver-Operator curves for significantly differentially expressed plasma cytokines. Area under curve (AUC) are shown in the graphs with one-sample Wilcoxon rank sum exact tests. Cytokines found significant or trending up in Figure 1A are displayed as following, IL1β (solid pink line), IL4 (green dotted line) and IL6 (blue dashed line) for each pair of groups.

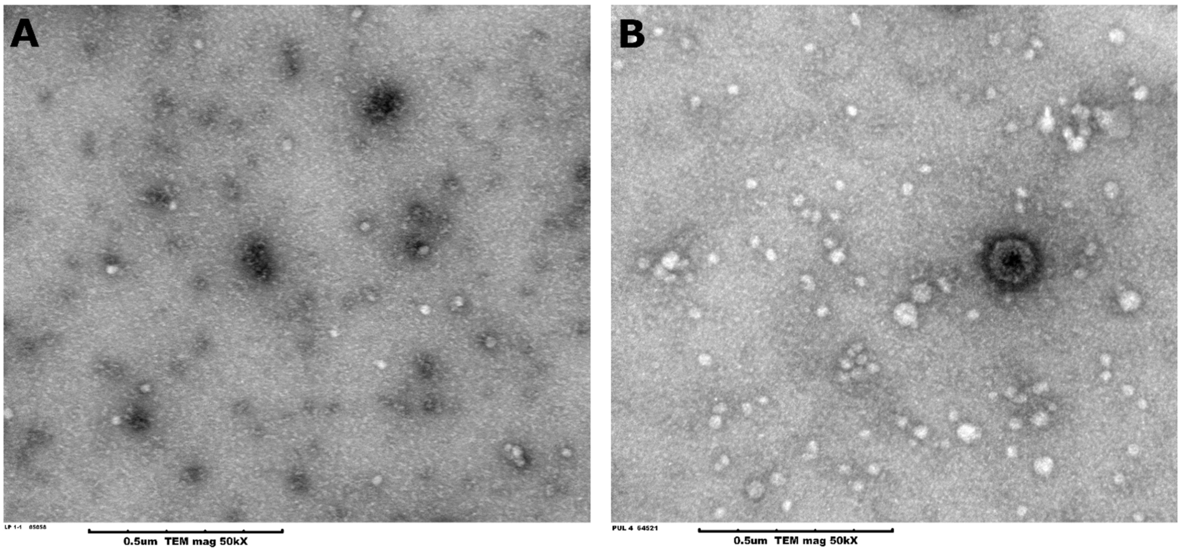

Figure S2. Transmission electronic microscopy of nEVs from plasma. The control nEVs are homogeneous (A) while the nEVs from the CoV (not shown) and nCoV (pictured) groups are heterogenous in size (B).
